# Measuring the uptake of continuous care among people living with HIV receiving antiretroviral therapy and social determinants of the uptake of continuous care in the southwest of China: a cross-sectional study

**DOI:** 10.1186/s12879-021-06644-0

**Published:** 2021-09-11

**Authors:** Yongmei Jin, Sawitri Assanangkornchai, Meiqin Fang, Wei Guan, Bo Tian, Min Yu, Yingrong Du

**Affiliations:** 1 Department of Infectious Diseases, The Third People’s Hospital of Kunming City, Kunming, Yunnan People’s Republic of China; 2 Epidemiology Unit, Faculty of Medicine, Prince of Songkla University, Hat Yai, Songkhla Thailand

**Keywords:** Human immunodeficiency virus, Healthcare utilization, Behavioral model for vulnerable populations, Principal component analysis, Ordinal logistic regression

## Abstract

**Background:**

Continuous care is essential for people living with HIV. This study aimed to measure continuous care uptake and investigate the association between higher uptake of continuous care and behavioral and social factors, including HIV-acquisition risk and socioeconomic characteristics.

**Methods:**

A hospital-based cross-sectional study was conducted from April to November 2019 in an HIV treatment center of a specialized hospital in Kunming city, China. Fourteen service indicators were used to calculate composite care scores, which were classified into three levels (low, middle, and high), using principal component analysis. The Behavioral Model for Vulnerable Populations was employed to examine predisposing, enabling, and need factors associated with composite care scores among people living with HIV.

**Results:**

A total of 702 participants living with HIV aged ≥ 18 years (median age: 41.0 years, 69.4% male) who had been on ART for 1–5 years were recruited. Based on ordinal logistic regression modeling, predisposing factors: being employed (adjusted odds ratio (AOR): 1.54, 95% confidence interval (CI): 1.13–2.11), heterosexuals (AOR: 1.58, 95% CI: 1.11–2.25) and men who have sex with men (AOR: 2.05, 95% CI: 1.39–3.02) and enabling factors: Urban Employee Basic Medical Insurance (AOR: 1.90, 95% CI: 1.03–3.54), middle socioeconomic status (SES) (AOR: 1.42, 95% CI: 1.01–2.01), were positively associated with the higher level of continuous care uptake, compared to the unemployed, people who inject drugs, those with no medical insurance and low SES, respectively.

**Conclusion:**

There were large differences in continuous care uptake among people living with HIV. HIV-acquisition risk categories and socioeconomic factors were significant determinants of uptake of continuous care. Our findings could inform the development of evidence-based strategies that promote equitable healthcare for all people living with HIV.

## Background

With a dramatic increase in survival time among people living with HIV due to antiretroviral therapy (ART), healthcare systems face new challenges in maintaining them in care and meeting their care needs [1, 2]. As the number of people receiving life-long ART continues to grow, the focus of HIV care has gradually transformed from urgent care to chronic care with regular routine management, similar to that for other systemic chronic conditions, in order to protect the person's physical and mental health [3–5]. People with HIV are living longer and aging, and have a more significant burden of comorbidities from chronic noncommunicable diseases (NCDs) than other people [6]. To address this challenge in HIV care, it is increasingly recognized that comprehensive multi-system screening and management integrated with HIV care has benefits for both adults living with HIV who are receiving care and the healthcare system [6–9].

Unlike access to care or engagement in HIV care, which only reflects the snapshot of reach care or use of HIV care, uptake of continuous care among people living with HIV means utilizing comprehensive care by those people over a certain time period, such as one year. Nevertheless, continuous care represents a new area of challenge in HIV care. Continuous care for people living with HIV receiving long-term ART involves scheduled visits and regular follow-up to monitor ART response, aligned with long-term ART. Infectious complications of HIV, such as tuberculosis, viral hepatitis, and some sexual transmitted infections, also continue to affect individuals' quality of life; screening and management of these complications are areas of focus [10–13]. Furthermore, HIV infection is related to some NCDs, including cardiovascular diseases, atherosclerosis, osteoporosis, and renal diseases [13–15]. With lifelong use of ART, the side effects of some of the medicines are also associated with non-HIV-related chronic conditions, including but not limited to alterations in metabolism, liver and renal functions [16], bone mineral density (BMD) [17], and neuropsychiatric effects [18]. Following the recommendation by current international guidelines for HIV treatment and prevention [10–12], continuous and comprehensive care for people living with HIV receiving ART involves a series of services, including regular follow-up visits to monitor their responses, management of HIV advanced diseases, comprehensive multi-system screening and management of coinfections and chronic complications, such as tuberculosis, viral hepatitis, syphilis, cardiovascular diseases, changes of liver and renal function and loss of BMD, which are associated with HIV infection, aging and access to ART [3, 19, 20]. Furthermore, routine assessment and management of mental health conditions, especially depression, are recommended to be integrated into the package of HIV care services for all people living with HIV in non-psychiatric healthcare settings [11, 21].

While continuous care is vital for people living with HIV, especially those who are stable and have been receiving ART for more than one year, less research is being done to explore utilization of continuous care. Studies have only assessed accessing and utilizing some HIV services within continuous care, especially among key populations such as people who inject drugs and men who have sex with men [22, 23], but little is known about individual and specific factors that influence the uptake of continuous care among people living with HIV receiving ART. Previous studies have indicated that some factors, including HIV-acquisition risk, socioeconomic status (SES), age, area of residence, health insurance, social support, and stigma, were associated with HIV epidemics, testing, maternal care, delays in treatment, access to ART and mental healthcare, attrition of ART, and even poorer treatment outcomes [23–28]. People living with HIV from a disadvantaged social class are more likely to have a delayed diagnosis, worse access to healthcare, and even worse treatment outcome, which are related to factors such as unemployment, low education, and poor living conditions [29]. People who inject drugs and other socially disadvantaged and marginalized populations generally suffer late diagnosis and initiate ART in the late stages of infection [30], compared to MSM who usually initiate ART earlier and are more likely to access HIV-related care, have higher adherence to ART, and experience better treatment outcomes [31]. Since HIV-acquisition risk has been demonstrated as a primary behavioral factor associated with HIV diagnosis and treatment, the effect of HIV-acquisition risk on uptake of continuous care among people living with HIV receiving ART should be carefully elucidated.

China also has a complicated HIV epidemic and faces challenges in managing a growing number of people living with HIV [32]. Reportedly, 861,042 surviving people were living with HIV in China in 2018 [33]. HIV epidemics in southwest China are interrelated across the three most at-risk populations [34], including people who inject drugs, people with unsafe heterosexual contact, and MSM [35, 36]. China now funds its national HIV responses almost entirely (98.4%) from domestic resources [37]. The National Free Antiretroviral treatment program (NFATP) was established in 2003 and rapidly expanded ART coverage in 2016 [1, 32]. The designated hospitals by the national programs are responsible for all components of HIV treatment and care. At the end of 2018, 718,499 people living with HIV (83.4%) in China were accessing ART through the NFATP [29]. Because of substantial attrition along the HIV care continuum, especially among people who inject drugs, the program faces severe challenges in retaining the increasing number of people living with HIV in care [1]. Some Chinese researchers focused on the importance of continuity in HIV care and the management of non-AIDS-related chronic complications, such as cardiovascular diseases, chronic kidney diseases, loss of BMD, highlighting the multidisciplinary chronic care needs of people living with HIV on lifelong ART [19, 20]. However, there is lack of consensus around comprehensive continuous care and the lack of certain interventions in the national Chinese guidelines. Current Chinese National Guidelines for HIV/AIDS Diagnosis and Treatment (2018 version) only focus on access to ART and monitoring response of ART, involving regular prescription of ART, annual monitoring of viral loads and CD4 counts, monitoring blood cell counts, liver function and urine tests, optional monitoring of renal function (optional for Tenofovir disoproxil fumarate), and blood lipids (optional for Lopinavir/Ritonavir) [38]. Multidisciplinary screening and management, an essential component of continuous and comprehensive care, as well as mental health care, is absent in the current Chinese guidelines.

Studies in China have shown income-related inequalities in linkage to care or attrition from treatment programs [23, 39]. These trends have been observed in the whole country as well as in regions including southwest China, the major site of the HIV epidemic [40]. However, there is a paucity of studies measuring continuous care for people living with HIV who are stable on ART as a whole, especially in the Asia–Pacific region. Moreover, under the context of free ART to treat all people living with HIV, no study has reported whether people living with HIV who have received ART are retained in the HIV care continuum and receive an equal amount of comprehensive care to maintain both their physical and mental health regardless of their socioeconomic characteristics and three main HIV exposure categories in China. Additionally, how suspected determinants affect the utilization of continuous care in southwest China necessitates a more in-depth examination. To address those gaps in the limited data on continuous care, the Behavioral Model for Vulnerable Populations was used to guide our approach [41]. It suggests that there are predisposing, enabling and need components that can predict the use of health services as well as the impact of utilization on health status outcome. This model has been used to understand other health service utilization among people living with HIV [42]. Such research is critical given the potential for continuous care on an HIV care modality. Identifying population groups with barriers to accessing ongoing comprehensive and continuous HIV care will apprise the development of interventions targeted towards the most vulnerable populations among people living with HIV.

This study was a part of a larger project aiming to examine continuous care patterns among people living with HIV who have been on ART in the southwest of China. The current study's first objective was to compare continuous care uptake among people with different exposure categories in Kunming city, southwest China. This was done by recruiting and characterizing people living with HIV according to three exposure categories as heterosexual men or women, men who have sex with men (MSM), and people who inject drugs, and comparing their uptake of continuous care elements, predisposing, enabling and need factors. The second objective was to develop a composite score of the uptake of all types of continuous care and identified the determinants of levels of continuous care uptake.

## Methods

### Study design and setting

We conducted a hospital-based cross-sectional survey from April to November 2019 at the Third People's Hospital of Kunming, the capital city of Yunnan province, southwest China. The hospital is the biggest hospital specializing in infectious diseases in Yunnan province, with a leading HIV treatment center, and has been serving people living with HIV since 1999. In 2004, as a designated center of the NFATP in Kunming, it launched a free ART program for people living with HIV and provided HIV care for local clients as well as those referred from other cities of China. As of 2018, this center has offered free ART and follow-up service for about 8,000 people.

Participants' eligibility criteria were (1) having self-reported exposure to HIV-acquisition risk as heterosexual contact, male homosexual contact, or injecting drugs use, (2) being aged 18 years or more, and (3) having been on ART for at least one but no more than five years. This last criterion was set in order to reach a requirement on the average time of retention in care to viral suppression and also to avoid some of the longer-term complications after five years, which need more intensive care [43–45]. Potential participants were assessed to be capable of participating in the study if they were physically and mentally able to provide verbal consent to participate in the study on the day of the interview.

### Study sample and procedures

A total sample size of 664 was first calculated to determine the rate of receiving continuous care among people living with HIV based on the assumption that such a rate was 50% and using a margin of error of 4%, a confidence interval (CI) of 95%, and a 20% refusal rate or missing data. To compare uptake of care among three exposure categories, we used the two independent proportions formula [46], and we assumed that the uptake rate among the reference group (people who inject drugs) was 50% and the uptake rate among one of the other groups was 65%. Given a ratio of 1:1, 80% power, and a 20% refusal rate, a minimum of 213 participants per group with different HIV-acquisition risks was required.

Using convenience sampling, all consecutive eligible people living with HIV who visited the HIV treatment center during the study period were recruited. When the minimum sample size of each HIV-acquisition risk group was reached, recruitment of subjects in the corresponding group stopped.

Recruitment took place over eight months with a 99.0% response rate. If a potential participant indicated an interest in participating, the hospital staff then referred them to one of our five trained research assistants who conducted the interview using a structured guideline. A structured questionnaire was developed in English and then translated into Chinese. Back translation into English was also performed to validate the translation. Pre-testing of the questionnaire was completed with twenty persons living with HIV not involved in the study to ensure the comprehensibility of the questionnaire. The research assistant informed the participants of the study goals, benefits and risks of participation, and research procedures. The participants were told that their responses would be anonymous. They were also informed that they could withdraw at any time, and all information would be kept confidential. With the verbal consent of the respondents, one-to-one interviews were conducted. Each participant provided their NFATP unique personal identification number in order to link their questionnaire data to their medical records. After completing the interview, the field supervisor reviewed the participants' medical records in the database of the NFATP and the outpatient system of the study hospital to collect information about the utilization of continuous care.

### Measures

#### Data source

Participants self-completed a set of questionnaires comprising demographic and socioeconomic information, perceived HIV-related stigma, perceived social support, other characteristics of accessibility to care, uptake of mental healthcare and diagnosis of chronic comorbidities in the last 12 months. Medical records in the database of the NFATP and outpatient system of the treatment center were reviewed to collect information about participant's routine follow-up and testing, infectious comorbidities such as viral hepatitis, and non-infectious chronic comorbidities generally associated with aging such as cardiovascular diseases, chronic kidney disease, and loss of bone mineral density during the last 12 months.

#### Indicators of continuous care

We developed indicators and domains of HIV continuous care based on existing literature and published guidelines (Table 1) [11, 12, 38, 47]. Based on China's free ART manual, subsequent follow-up visits are scheduled for every three months among people who are stable on ART [48]. With the central government's financial support, routine blood tests accompanied by follow-up visits four times a year and at least one viral load test and CD4 count per year are free for people who are stable on ART [48]. Follow-up indicators include the frequency of HIV clinic visits and free routine follow-up testing. Routine ART monitoring involved frequency of CD4 testing and viral load assay while extra multi-system screening included risk of cardiovascular [49] and chronic kidney diseases [50], electrocardiogram (ECG), assessment of bone mineral density [20], tuberculosis screening, and serological testing for hepatitis B and C viruses and syphilis during the past 12 months. Except for BMD, which was measured in another tertiary hospital, all HIV-related care was carried out in the study hospital. Laboratory tests were arranged at the same clinic visit as much as possible. BMD was not free of charge and measured by dual-energy X-ray absorptiometry scanner, which is the gold standard for the diagnosis of osteoporosis according to the WHO [51].

**Table 1:** Summary of indicators used to create the composite score of continuous care uptake for people living with HIV on antiretroviral therapy

Indicator	Source	Definition	Goal
Clinic visit	NFATP	Frequency of patient's visits at an HIV clinic in the past six months for ART drug refill	Two or more in the past six months
Routine test	NFATP	Frequency of respondent's free routine testing including simple renal function (i.e., plasma creatinine, urea), liver function (i.e., alanine aminotransferase and aspartate aminotransferase), complete blood cell counts in the last 12 months for monitoring toxicity of ART	Four or more in the past 12 months
CD4 count	NFATP and outpatient system	Frequency of respondent's CD4 testing in the last 12 months for immunological monitoring	For counts between 300 and 500 cells/mm^3^, receiving at least one test in the last 12 months, and for counts less than 300 cells/mm^3^, receiving at least two tests in the last 12 months [12, 34]
Viral load assay	NFATP and outpatient system	Frequency of respondent's viral load assay in the last 12 months for virological monitoring	At least twice in the last 12 months [12, 34]
Mental health assessment	Self-report	Respondent's answer to the question "Have you received a mental health assessment from a health provider in the last 12 months?" with "Yes" or "No"	Received in the last 12 months
Mental health counseling	Self-report	Respondent's answer to the question "Have you received mental health counseling from a health provider in the last 12 months?" with "Yes" or "No"	Received in the last 12 months
BMD	Outpatient system	Whether the respondent had a lumbar spine and femur BMD by dual-energy X-ray absorptiometry scanner in the last 12 months	Received in the last 12 months
Evaluation of CKD risk	Outpatient system	Whether the respondent received evaluation of the risk of CKD, including calculation of creatinine clearance (Cockcroft-Gault' formula), urinalysis with urine glucose, urine protein and urinary β 2-microglobulin in the last 12 months	Received in the last 12 months
Monitoring CVD risk	Outpatient system	Whether the respondent received measurement of blood lipids and blood pressure for monitoring risk of CVD in the last 12 months	Received in the last 12 months
ECG	Outpatient system	Whether the respondent had an ECG in the last 12 months	Received in the last 12 months
TB screening	Outpatient system	Whether the respondent had a chest X-ray and further sputum smear in the last 12 months	Received in the last 12 months
Hepatitis B serologic testing	Outpatient system	Whether the respondent had testing of HBV antigen and antibody, or HBV-DNA in the last 12 months	Received in the last 12 months
Hepatitis C serologic testing	Outpatient system	Whether the respondent had testing for anti-HCV, HCV-RNA, or other HCV genotype in the last 12 months	Received in the last 12 months
Syphilis serological testing	Outpatient system	Whether the respondent had testing a nontreponemal test (i.e., RPR) and a treponemal test (i.e., the TP-PA) in the last 12 months	Received in the last 12 months

*BMD* bone mineral density, *CKD* chronic kidney diseases, *CVD* cardiovascular diseases, *ECG* electrocardiogram, *TB* tuberculosis, *HBV/HCV* hepatitis B/C virus, *NFATP* National Free Antiretroviral treatment program, *RNA* Ribonucleic acid, *DNA* Desoxyribonucleic acid, *RPR* rapid plasma regain, *TP-PA* T. pallidum passive particle agglutination assay

#### Computation of composite score of continuous care

**Fig. 1:**
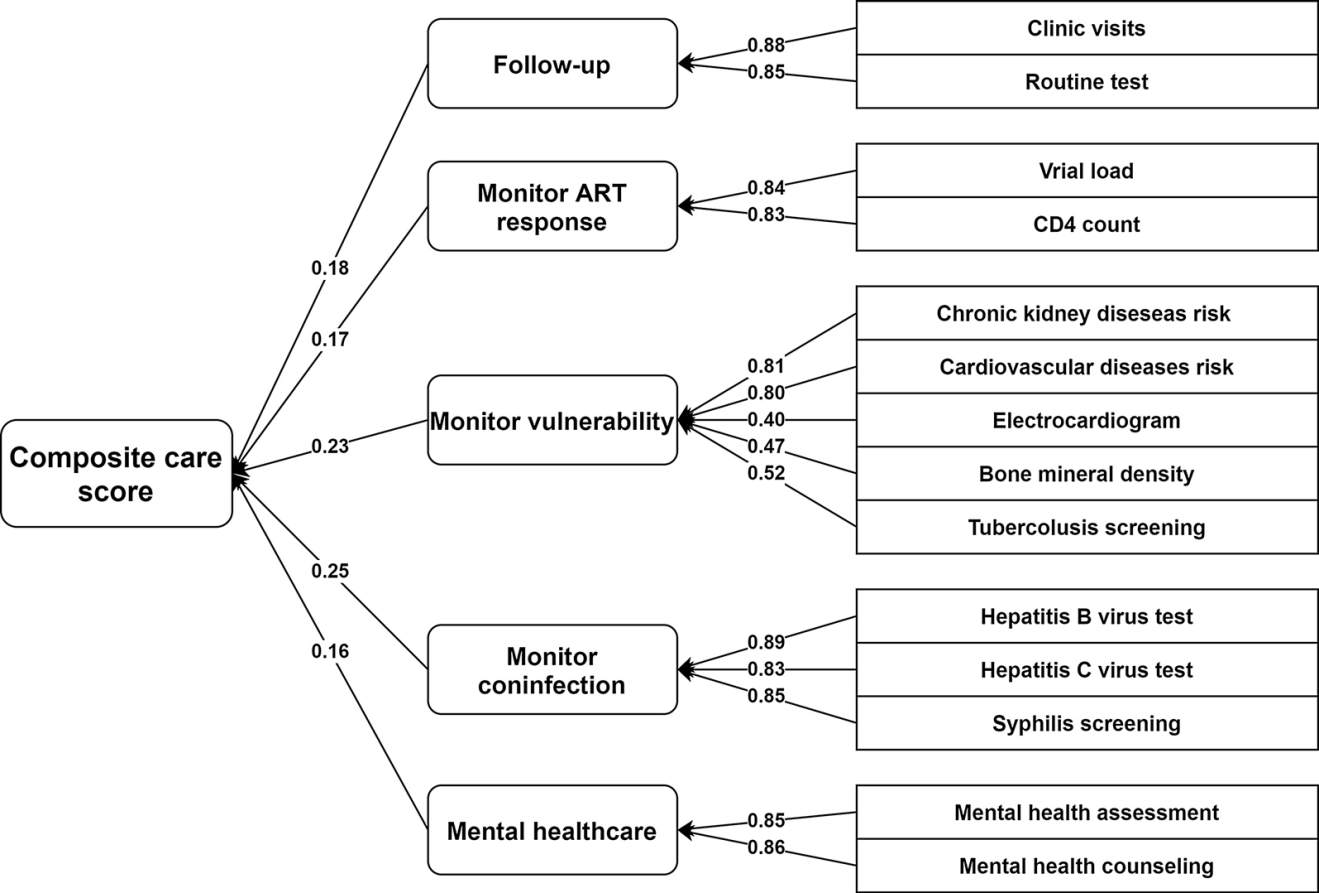
Conceptual framework for the composite score of continuous care for people living with HIV

The comprehensive care package includes 14 indicators reflecting continuous care for people living with HIV who are on ART. We developed a composite score using principal component analysis (PCA), which reduces the number of dimensions in high-dimensional data while retaining most of the features and patterns [52]. The optimal number of dimensions based on a scree plot was five. Weights were derived from these five components that collectively accounted for 79.6% of the variance, with the first to fifth components explaining 25%, 23%, 28%, 17%, and 16% of the variance, respectively (Fig. 1). Using varimax rotation, we condensed the 14 indicators into five principal components relevant to each dimension, and an initial score (IS) was formulated by summing the product of each of those component weights with their corresponding PCA scores [53].

The value of the initial score can be positive or negative, making it difficult to interpret. Therefore, a standardized composite score (CS) was developed using the formula:

1 $${\text{CS }} = { 1}00 \, \times \, \left( {{\text{IS }} - {\text{ minimum IS}}} \right) \, / \, \left( {{\text{maximum IS }} - {\text{ minimum IS}}} \right)$$

Higher values of the standardized composite score reflect higher uptake of continuous care. The median score was 45.0 with an interquartile range (IQR) of 36.4 to 51.7 and a range of 0 to 100. The outcome of interest was continuous care, classified into terciles of low, middle, and high based on the standardized score.

#### Study explanatory variables

To examine the factors that influence the uptake of continuous care, we employed the Behavioral Model of Health Services Utilization for Vulnerable Populations [41] in which the factors can be categorized into three domains of explanatory variables as follows:

##### Predisposing factors

These factors refer to characteristics that people possess and influence the propensity of individuals to seek care. Traditional predisposing factors were age (18–34, 35–49 and 50 + years), sex, ethnicity (Han and other), religious beliefs (with or without), marital status, education level (primary school or below, secondary school, high school, and university or equivalent), employment status (employed and unemployed), vulnerable predisposing factors of HIV-related stigma and HIV-acquisition risk. HIV-related stigma was assessed using a Chinese version of internalized stigma and personal stigma scales [54]. HIV-acquisition risk was derived from the database of the NFATP and classified into three main categories: heterosexual contact, homosexual contact, and injecting drug use [29, 55]. Unlike in Western countries, the epidemic of HIV among MSM in China indicated that there is almost no interaction between risk groups of MSM and people who inject drugs or, at least, there is very limited crossover [56], so we distinguished MSM and those who also inject drugs into different groups.

##### Enabling factors

These factors refer to the resources that enable the utilization of care. In this study, we focused on the area of residence (rural, urban, suburban), socioeconomic status, social support, type of medical insurance (uninsured, New Rural Cooperative Medical Insurance (NRCMI), Urban Employees Basic Medical Insurance (UEBMI), and Urban Residents Basic Medical Insurance (URBMI)) and the accessibility to care (e.g., distance to nearest HIV facility, travel time, transportation fee, waiting time for medicine). Socioeconomic status was measured by a wealth index, which was generated from self-reported household assets and household conditions as reported elsewhere [57] and categorized into terciles (low, middle, and high). Social support (number of people) was measured using the Social Support Questionnaire (SSQ) [58].

##### Need factors

These factors describe the need for health care determined by individual perception or professional medical evaluation, which are direct causes of the use of medical services. We included the presence of chronic comorbidities based on the Charlson Comorbidity Index and ART duration. Besides AIDS, the Charlson Comorbidity Index accounts for eleven chronic comorbidities, including diabetes mellitus, liver disease, malignancy, chronic kidney disease, congestive heart failure, myocardial infarction, chronic obstructive pulmonary disease, peripheral vascular disease, cerebrovascular disease or transient ischemic attack, connective tissue disease, and peptic ulcer disease [59]. We employed a checklist of these eleven comorbidities in both the questionnaire and medical records review. Chronic comorbidity was defined as having at least one of these chronic diseases identified from either the questionnaire or medical record. ART duration was obtained from the database of NFATP, which was defined as the time since ART initiation in years.

### Statistical analysis

We performed all analyses in R version 4.0.3 using descriptive statistics to summarize participant characteristics and considering a p-value less than 0.05 as statistically significant. Mean and standard deviation (SD) were used to describe normally distributed continuous variables, median with IQR for non-normally distributed variables, and frequency and percentage for categorical variables. The Kruskal–Wallis test was used to compare the distribution of a non-normal continuous variable among groups. The Chi-square test was used to compare the difference in exposure of HIV-acquisition risk and categorical groups of care uptake. The probabilities of dependent variables in different age groups, education levels, and SES groups were calculated and compared using the multinomial Cochran-Armitage trend test with Bonferroni's correction for multiple comparisons. Due to the ordinal nature of care uptake from the composite score, ordinal logistic regression using a proportional odds model was used to investigate the effects of the determinants of higher uptake. Before modeling, we employed the omnibus Brant test for testing the parallel line regression assumption for the proportional odds assumption. A non-significant p-value for the full model and final model indicates that the proportional odds assumption for the model was tenable. The univariate and multivariate proportional odds models were fitted with low uptake as the baseline outcome level. We further performed a backward stepwise multivariate ordinal logistic regression model and eliminated variables that were not significant in the univariate model (p-value > 0.05). An ordinal odds ratio (OR) with a 95% confidence interval (CI) was presented.

## Results

### Socio-demographic characteristics of people living with HIV on ART

A total of 702 adults with HIV on ART (heterosexuals, MSM, and people who inject drugs comprise one-third of the sample) attending the HIV treatment center in Kunming participated in the study. The age of the participants ranged from 18 to 77 years with a median age of 41 years. MSM had significantly higher percentages of participants aged between 18–34 years, employed, with university or higher education and middle to high SES. Basic medical insurance covered 92.7% of participants. The scores of HIV-related stigma were similar among the three groups, but MSM had significantly higher social support (Table 2).

**Table 2:** Socio-demographic characteristics of people living with HIV on ART by HIV-acquisition risk category

Characteristic	Total (n = 702)	HIV-acquisition risk category			p-value
		Heterosexuals (n = 239)	MSM (n = 231)	PWID (n = 232)	
Age (years)					< 0.001^††^
18–34	34.8	25.1	70.6	9.1	
35–49	46.2	43.1	24.2	71.1	
50 +	19.1	31.8	5.2	19.8	
Sex					< 0.001
Male	69.4	43.1	100.0	65.9	
Female	30.6	56.9	0.0	34.1	
Ethnicity					0.505
Han	83.9	85.8	81.8	84.1	
Other	16.1	14.2	18.2	15.9	
Religious belief					0.107
With	16.1	13.8	14.3	20.3	
Without	83.9	86.2	81.8	79.7	
Marital status					< 0.001
Married	44.3	64.9	19.9	47.4	
Single	35.3	8.4	73.2	25.4	
Divorced	15.7	17.2	6.9	22.8	
Widowed	4.7	9.6	0.0	4.3	
Residence					< 0.001
Rural	9.8	10.0	6.5	12.9	
Urban	75.2	78.7	81.4	65.5	
Suburban	15.0	11.3	12.1	21.6	
Employment status					< 0.001
Unemployed	44.9	46.4	18.6	69.4	
Employed	55.1	53.6	81.4	30.6	
Education level					< 0.001
Primary school or below	24.8	39.7	6.1	28.0	
Secondary school	34.9	35.1	17.7	51.7	
High school	17.7	15.1	21.6	16.4	
University or equivalent	22.6	10.0	54.5	3.9	
Medical insurance					< 0.001
Uninsured	7.3	6.3	7.4	8.2	
NRCMI	44.3	59.0	33.8	39.7	
UEBMI	17.9	11.7	36.4	6.0	
URBMI	30.5	23.0	22.5	46.1	
SES					0.037^††^
Low	33.3	36.4	25.5	37.9	
Middle	33.3	31.6	37.2	31.0	
High	33.3	31.8	37.2	31.0	
HIV-related stigma	44.0 (41.0, 47.0)	44.0 (41.0, 47.0)	44.0 (41.0,46.5)	45.0 (41.0, 47.2)	0.071
SSQN	1.2 (0.7, 2.0)	1.0 (0.5, 1.8)	1.7 (0.8, 2.5)	1.0 (0.5, 2.0)	< 0.001

Data are column percentage or median (IQR). *MSM* men who have sex with men, *PWID* people who inject drugs, *NRCMI* New Rural Cooperative Medical Insurance, *UEBMI* Urban Employees Basic Medical Insurance, *URBMI* Urban Residents Basic Medical Insurance, *SES* socioeconomic status. SSQN, social support questionnaire number. ^††^Multinomial Cochran-Armitage trend test

### Profile of uptake of continuous care and care-related accessibility among people living with HIV on ART

Table 3 describes the percentages of each indicator of uptake of continuous care. Most indicators had high rates of uptake (63.5 – 93.3%), with exceptions being adequate virological monitoring (15.8%), mental health assessment (15.0%) and counseling (30.8%), and BMD measurement (36.5%).

**Table 3:** Uptake of indicators of continuous care and other characteristics of accessibility of care for people living with HIV on ART according to HIV-acquisition risk

	Total (n = 702)	HIV-acquisition risk category			*p* value
		Heterosexuals (n = 239)	MSM (n = 231)	PWID (n = 232)	
Indicators of continuous care (%)					
Adequate routine visit	82.9	77.0	87.9	84.1	0.006
Adequate routine test	81.9	80.8	85.7	79.3	0.171
Adequate CD4 testing	80.6	80.8	87.0	74.1	0.002
Adequate viral load testing	15.8	15.9	23.8	7.8	< 0.001
Mental health assessment	15.0	15.9	23.8	7.8	< 0.001
Mental health counselling	30.8	20.9	31.6	40.1	< 0.001
BMD measurement	36.5	40.2	46.3	22.8	< 0.001
CKD risk evaluation	84.8	91.6	94.4	68.1	< 0.001
CVD risk monitoring	84.6	93.7	89.2	70.7	< 0.001
ECG	71.1	74.5	71.0	67.7	0.265
TB screening	93.3	98.7	95.7	85.3	< 0.001
HBV serological test	63.5	62.8	73.6	54.3	< 0.001
HCV serological test	70.4	59.4	68.4	83.6	< 0.001
Syphilis serological test	51.0	53.1	60.6	39.2	< 0.001
Other characteristics of accessibility of care					
Chronic comorbidities (%)	39.2	18.0	13.0	87.1	< 0.001
Distance to HIV facilities, (kilometers)	10.0 (5.0,30.0)	11.0 (7.0,40.0)	10.0 (5.0, 20.0)	10.0 (5.0, 30.0)	0.005
Travel time, (hour)	0.7 (0.5,1.0)	1.0 (0.5,1.5)	0.5 (0.5,1.0)	0.7 (0.3,1.0)	< 0.001
Transportation fee, (yuan)	6.0 (2.2,20.0)	6.0 (2.0,23.5)	10.0 (3.0,20.0)	5.0 (2.0,20.0)	0.310
Waiting time for getting medicine, (hour)	1.0 (0.7,2.0)	1.5 (1.0, 2.0)	1.5 (1.0, 2.0)	1.0 (0.5,2.0)	< 0.001
ART duration, (years)	3.7 (2.1, 4.7)	3.8 (2.2, 4.6)	3.0 (1.6, 4.4)	4.1 (2.7, 4.7)	< 0.001

Data are column percentage or median (IQR). *MSM* men who have sex with men, *PWID* people who inject drugs, *CKD* chronic kidney diseases, *CVD* cardiovascular diseases, *TB* tuberculosis, *ECG* electrocardiogram, *HBV/HCV* hepatitis B/C virus

Significant differences among the three groups were observed for uptake of continuous care and accessibility of care indicators. MSM had the highest proportion of uptake in all care indicators, except for the hepatitis C virus serological test. People who inject drugs accounted for the highest proportion (87.1%) of being diagnosed with chronic comorbidities (p < 0.001). There were also significant differences in the distance to the nearest HIV facility, travel time, and waiting time for receiving medicine. ART duration among the three groups was significantly different with men who have sex with men having a lower duration than the other two groups. No substantial differences in the adequate uptake of routine tests, electrocardiograms, and transportation fees to HIV facilities were observed.

### Determinants of uptake of continuous care for people living with HIV on ART

Based on the univariable ordinal logistic regression analysis, age, gender, employment status, medical insurance, education level, SES, HIV-acquisition risk, chronic comorbidities, and the number of social supporters were significantly associated with the composite scores of continuous care. However, there were no significant differences in the composite care scores by characteristics of accessibilities to care, including distance to nearest HIV facility, travel time, transportation fee, waiting time for medicine, and treatment duration (Table 4).

**Table 4:** Distribution of scores for uptake of continuous care and results of univariable ordinal logistic regression analysis (unadjusted) according to level of composite care score of continuous care

Characteristic	Level of composite care (%)			p-value	Unadjusted ordinal OR (95% CI)
	Low (n = 234)	Middle (n = 235)	High (n = 233)		
Age (years)				0.056^††^	
18–34	26.9	35.9	41.5		Ref.
35–49	53.8	43.2	41.5		0.60 (0.44–0.81)**
50 +	19.2	20.9	17.1		0.69 (0.47–1.02)
Sex				0.016	
Male	60.3	73.1	74.8		Ref.
Female	56.9	0.0	34.1		0.60 (0.45–0.81)**
Ethnicity				0.089	
Han	82.5	82.1	87.2		Ref.
Other	17.5	17.9	12.8		0.78 (0.54–1.12)
Religious belief				0.305	
With	14.1	17.1	16.1		Ref.
Without	85.9	82.9	82.9		0.83 (0.57–1.19)
Marital status				0.522	
Married	47.0	42.7	43.2		Ref.
Single	32.1	36.8	37.2		1.22 (0.90–1.65)
Divorced	17.1	17.9	14.5		1.14 (0.77–1.69)
Widowed	6.4	2.6	5.1		0.87 (0.43–1.74)
Residence				0.237	
Rural	9.8	9.8	9.8		Ref.
Urban	70.9	77.4	75.2		1.06 (0.67–1.69)
Suburban	19.2	12.8	12.8		0.72 (0.41–1.26)
Employment status				< 0.001	
Unemployed	58.5	43.2	32.9		Ref.
Employed	41.5	56.8	67.1		2.20 (1.66–1.80)***
Education level				< 0.001^††^	
Primary school or below	29.9	26.5	17.9		Ref.
Secondary school	44.0	28.6	32.1		1.12 (0.78–1.60)
High school	13.2	20.5	19.2		1.92 (1.26–2.94)**
University or equivalent	12.8	24.4	30.8		2.67 (1.79–3.98)***
Medical insurance				< 0.001	
Uninsured	7.3	9.0	5.6		Ref
NRCMI	49.6	43.6	39.7		1.05 (0.61–1.80)
UEBMI	6.4	20.9	26.5		2.89 (1.60–5.26)**
URBMI	36.8	26.5	28.2		0.99 (0.57–1.72)
SES				0.060^††^	
Low	38.5	35.0	25.6		Ref.
Middle	28.2	33.8	38.0		1.63 (1.17–2.28)**
High	33.3	30.3	36.3		1.44 (1.03–2.01)*
HIV-acquisition risk				< 0.001	
PWID	47.4	27.8	23.9		Ref.
Heterosexuals	35.0	33.8	38.0		1.63 (1.17–2.28)**
MSM	17.5	38.5	42.7		3.10 (2.20–4.39)***
Chronic comorbidities				< 0.001	
Yes	51.3	35.9	30.3		Ref.
No	48.7	64.1	69.7		1.98 (1.49–2.63)***
HIV-related stigma	44.5 (42.0–47.0)	44.0 (41.0–47.0)	44.0 (41.0–47.0)	0.526^†^	0.98 (0.96–1.01)
Social support	1.2 (0.3–2.0)	1.3 (0.8–2.3)	1.2 (0.7–2.0)	0.007^†^	1.12 (1.01–1.23)*
Distance to HIV facility, (km)	10.0 (5.0–30.0)	10.0 (6.0–24.5)	10.0 (5.0–30.0)	0.198^†^	1.00 (0.99–1.00)
Travel time, (hours)	1.0 (0.5–1.0)	0.5 (0.4–1.0)	0.7 (0.5–1.0)	0.405^†^	1.01 (0.93–1.09)
Transportation fee, (yuan)	8.0 (2.0–20.0)	6.0 (2.0–21.5)	6.0 (2.0–20.0)	0.834^†^	1.00 (0.99–1.00)
Waiting time for medicine, (hour)	1.5 (1.0–2.0)	1.0 (0.7–2.0)	1.0 (0.7–2.0)	0.217^†^	0.99 (0.90–1.10)
ART duration, (years)	3.8 (2.2–4.6)	3.7 (2.2–4.7)	3.7 (2.1–4.7)	0.905^†^	0.99 (0.90–1.10)

Data are column percentage or median (IQR). *SES* socioeconomic status, *NRCMI* New Rural Cooperative Medical Insurance, *UEBMI* Urban Employees Basic Medical Insurance, *URBMI* Urban Residents Basic Medical Insurance, *PWID* people who inject drugs, *MSM* men who have sex with men, *IDU* injecting drug use, *Ref.* reference group. ^†^Kruskal–Wallis test. ^††^Multinomial Cochran-Armitage trend test. ***, **, *Significant at levels 1, 5 and 10%, respectively

We included nine potential predictors of the higher composite scores of continuous care derived from univariate analyses in a multivariable ordinal logistic regression model (Table 5). Only four variables, employment status and HIV-acquisition risk (predisposing factors), medical insurance and SES (enabling factors), were retained in the final model, indicating significantly independent determinants of the higher composite scores of continuous care among people living with HIV. After the parallel line test, the final model did not violate the proportional odds assumption.

**Table 5:** Results of multivariable ordinal logistic regression analysis according to level of composite care score of continuous care

Characteristic	Adjusted ordinal OR (95% CI)	*p*-value
Employment status		
Unemployed	Ref.	
Employed	1.54 (1.13–2.11)	0.007
Medical insurance		
Uninsured	Ref.	
NRCMI	0.95 (0.55–1.66)	0.856
UEBMI	1.90 (1.03–3.54)	0.042
URBMI	1.07 (0.61–1.90)	0.818
SES		
Low	Ref.	
Middle	1.42 (1.01–2.01)	0.046
High	1.34 (0.95–1.90)	0.095
HIV-acquisition risk		
PWID	Ref.	
Heterosexuals	1.58 (1.11–2.25)	0.012
MSM	2.05 (1.39–3.02)	< 0.001

*SES* socioeconomic status, *NRCMI* New Rural Cooperative Medical Insurance, *UEBMI* Urban Employees Basic Medical Insurance, *URBMI* Urban Residents Basic Medical Insurance, *PWID* people who inject drugs, *MSM* men who have sex with men, *CI* confidence interval, *Ref.* reference group

Employed people had 1.54 times higher odds of having higher composite care scores (AOR: 1.54, 95% CI: 1.13–2.11) compared to unemployed people, holding constant all other variables. The tendency to reach higher composite care scores for people living with HIV was 1.58 (95% CI: 1.11–2.25) and 2.05 (95% CI: 1.39–3.02) times higher among heterosexuals and MSM, respectively, when compared with people who inject drugs. The cumulative odds for patients with UEBMI was 1.90 (95% CI: 1.03–3.54) times higher than the uninsured participants to attain a higher composite care score. Participants with a middle socioeconomic status level had 1.42 (95% CI: 1.01–2.01) times greater odds to receive a higher composite care score of continuous care than patients with a low socioeconomic status.

## Discussion

Large differences in continuous care uptake among people living with HIV in Kunming, China was observed, spanning from a low rate of 15% in mental health assessment to higher than 90% in tuberculosis screening. This finding is similar to studies from Vietnam and China [60, 61]. Compared with heterosexuals and people who inject drugs, MSM were more likely to receive a higher uptake of continuous care in terms of adequate clinic visits, adequate CD4 and viral load tests, mental health assessment, measuring bone mineral density, chronic kidney disease risk, hepatitis B virus and syphilis serological test. On the other hand, people who inject drugs were more likely to receive mental health counseling and get tested for the hepatitis C virus but less likely to attain other forms of care than MSM and heterosexuals. However, the signal indicator may not reflect the overall situation of comprehensive continuous care that people living with HIV need.

We constructed a novel measure of an individual's healthcare utilization—a composite care score—by using highly standardized estimates of 14 different types of care utilization. The composite care score provides a clearer signal on personal healthcare utilization and helps to account for variation due to behavior and social environment. Application of a composite score or index has been elaborated with broader implications for gauging utilization of complicated services or clinical indicators complex for a disease, evaluating the efficiency of the healthcare system, identifying disparities, improving quality of care, and allocating healthcare resources [62, 63].

In the predisposing domain, employed people living with HIV were more likely to receive higher levels of care, probably because of their higher SES and/or medical insurance. Employment can reduce people's financial hardships and sustain their personal financial resources and has been found to be associated with accessing HIV care [64]. Further, based on a composite care score, we found that MSM and heterosexuals were more likely to receive higher levels of continuous care than people who inject drugs. Our result adds to the literature that exposure of HIV-acquisition risk is an important independent predisposing determinant of healthcare utilization for people living with HIV. Consistent with previous studies worldwide, intravenous drug use was identified as a risk for HIV late diagnosis, discontinuity of HIV care, access to care or ART, poor treatment outcomes, and even less survival time for people who inject drugs [1, 31, 65, 66]. People who inject drugs are recognized as one of the key populations who often suffer from punitive laws or stigmatizing policies or are most at risk of acquiring or transmitting HIV [67]. Thus, their engagement in HIV care is important to a successful HIV response around the world. Healthcare settings may provide an environment that reinforces the care-seeking behaviors of people who inject drugs and their retention in care. The finding of different care uptake levels among people living with HIV with different exposure of HIV-acquisition risk suggests that healthcare providers should take into account the specific needs and situation of all people in the population to ensure equitable healthcare. Engaging people living with HIV to better understand their healthcare needs, including those who are not in care or receive less care than they require, could aid the development of more effective and efficient strategies for universal health coverage.

Among enabling factors in the Behavioral Model, the middle SES group was significantly associated with composite care scores with participants in the middle SES group being 1.42 times more likely to receive a higher level of continuous care than those with a low SES. This result is consistent with current global thinking around the bidirectional relationship between socioeconomic-related inequality and poor utilization of HIV care [25]. Therefore, the allocation of healthcare resources should put more concern on poor or vulnerable people. Interestingly, participants with a high SES were not more likely to receive more continuous care. This may be because this subgroup of people may prefer to have their services in a private setting or other general hospitals where information could not be collected. Unsurprisingly, the enabling factor of medical insurance had a significant effect on the uptake of continuous care. In this study, people with urban employee medical insurance had a higher likelihood of receiving higher levels of care than those with other types of medical insurance. This study confirmed a previous study [68] indicating dual vulnerability challenges and lack of opportunities to make better choices due to limited employment and health insurance among the poor or the unemployed. Although China’s national HIV care program provides free general laboratory tests, CD4 and viral load tests once a year, people living with HIV have to pay for the remaining tests and examinations, such as BMD measurements, additional viral load tests, and serological tests of viral hepatitis. The tests which are not free were voluntarily taken based on the client’s ability to pay, and test costs can be beyond their affordability. This may explain the relationships between composite care scores and socioeconomic status, medical insurance and employment status, leaving alone other potential financial barriers (transport costs and busy working).

Without interventions, socio-demographic factors limiting access to HIV-related services, ART, and retention in care would undoubtedly jeopardize the ending of AIDS by 2030 [66].

Healthcare providers could play an essential role in reducing inequalities in healthcare utilization among people living with HIV by inquiring about additional social and structural barriers to care access. Continuous care for people living with HIV who were unemployed or had a low SES should also be given priority. Financial subsidies or increasing reimbursements of non-employee medical insurance, job referral and training in the community may be potential community interventions for comprehensive continuous care for people living with HIV [25]. Beyond clinic-level interventions aiming at developing equality of healthcare utilization, individual-level interventions such as enhanced awareness of viral load monitoring, management of mental health, and monitoring chronic disease risks should be offered with greater completeness and consistency to the identified vulnerable groups, particularly people who inject drugs, the unemployed, and people with low SES, after they have received ART. The fact that significant differences in uptake of continuous care in HIV exposure categories and socioeconomic characteristics existed, even after accounting for demographic characteristics and care-related accessibility, suggests that many factors that differentially influence utilization of continuous care remain. These should be the focus of future research and targeted interventions to provide fair and high-quality healthcare for all people living with HIV.

This study collected common and comprehensive services suggested for all people living with HIV [69, 70] and could be accessed easily in most hospitals in China. However, the findings of this study should be interpreted in the context of several limitations. Firstly, the results can be generalized only to people living with HIV who had been on ART for at least one year but less than five years. Secondly, a nonprobability sampling method was used to recruit study participants with different HIV-acquisition risks. This could lead to sampling bias as the distribution of the people with HIV with different HIV-acquisition risks in our clinic might not be similar to that of the general HIV population. Therefore, it could limit the generalizability of our findings to the general HIV population. Thirdly, there are some limitations related to the construction and use of the composite care scores. These scores employed PCA, which tends to minimize the contribution of each indicator. This may reduce the reliability of the composite care score because some indicators perform well in one study and others in another. This score may not truly reflect the quality of care given the heterogeneity in clients’ needs and test availability. However, PCA is the most credible and scientific approach for combing data from different indicators while generating weights for each dimension. This increases concern over the validity of composite care scores. Levels of care uptake were obtained by classifying the composite care scores and ranking the scores prior to grouping. The score only provided a relative measure of inequality in care uptake, it cannot provide information on absolute levels of care uptake. Fourthly, the wealth status defined in this study was classified into terciles to minimize incorrect inferences based on an extremely skewed socioeconomic distribution. We also found instances of over- and under-estimation of SES in some settings that are not reflective of reality. Fifthly, causality cannot be established due to the nature of our cross-sectional study design. Lastly, as a hospital-based study, there may be information bias because we did not collect information about those not registered in the study hospital or did not utilize care of the national program. There may be recall bias in the perceived use of mental health care; however, we used limited time of perceived use of mental health care in the last 12 months, which may somehow reduce recall bias.

## Conclusions

Based on a composite score to measure continuous care, we identified HIV-acquisition risk, employment, socioeconomic status, and medical insurance as independent determinants of receiving a higher uptake of continuous care among people living with HIV in the southwest of China. Findings could contribute to informing the development of need-oriented health resource allocation and evidence-based strategies that promote equitable healthcare.

## Data Availability

The data used in this study are available from the corresponding author on reasonable request.

## Abbreviations

AOR: Adjusted odds ratio
ART: Antiretroviral therapy
BMD: Bone mineral density
CI: Confidence interval
CKD: Chronic kidney diseases
CVD: Cardiovascular diseases
DNA: Desoxyribonucleic acid
ECG: Electrocardiogram
HIV: Human immunodeficiency virus
HBV/HCV: Hepatitis B/C virus
MSM: Men who have sex with men
NCDs: Noncommunicable diseases
NFATP: National Free Antiretroviral Treatment Program
NRCMI: New Rural Cooperative Medical Insurance
RPR: Rapid plasma regain
PCA: Principal component analysis
PWID: People who inject drugs
RNA: Ribonucleic acid
SES: Socioeconomic status
TB: Tuberculosis
TP-PA: *T. pallidum* Passive particle agglutination assay
UEBMI: Urban Employees Basic Medical Insurance
URBMI: Urban Residents Basic Medical Insurance
